# Self-Perceived Competence in Providing Eating Disorder Interventions for Young People: A Service Evaluation Project

**DOI:** 10.1192/bjo.2024.511

**Published:** 2024-08-01

**Authors:** Emily Rogers, Joshua Lusby, Olakunle Oginni

**Affiliations:** 1 Cardiff & Vale University Healthboard, Cardiff, United Kingdom; 2 Cardiff University, Cardiff, United Kingdom

## Abstract

**Aims:**

Despite the rising prevalence of eating disorders among young people and their associated morbidity and mortality, the level of self-perceived competence of professionals in providing health interventions is unknown. It could be expected that those with low self-perceived competence would be reluctant to initiate therapeutic interventions, which may increase the burden of unmet need for this population. Consequently, a service evaluation project was carried out in Cardiff and Vale Health Board Trust to assess the confidence of healthcare providers in working with young people with eating disorders, and to identify interventions acceptable to clinicians in order to meet this service need.

**Methods:**

Fifty-two healthcare workers who worked with young people below 18 years responded to a brief survey. The survey was advertised via email through the medical education department between December 7 2023 and January 5 2024 to healthcare workers based at Adult and Paediatric Emergency departments, in-patient units of General Adult Medical and Paediatric departments and the Community-based Child and Adolescent Mental Health Services (CAMHS). The survey elicited participants’ specialty, location of practice, self-assessed confidence in managing eating disorders in young people, aspect of management participants require support in, and preferred interventions which might support greater literacy in this topic.

**Results:**

Fifty-two participants responded to our survey of whom 48% (25) were doctors, 17% (9) were psychologists, and 13% (7) were nurses. The larger proportion of participants worked in CAMHS (42%) and Paediatric wards/emergency department (37%). About 43% reported having a role in managing young people with eating disorders. Half of the participants reported having “average” to “good” confidence in managing young people with eating disorders while none reported “very good” confidence. Discussion with colleagues was reported as the most common means of getting information about managing young people with eating disorders (79%), while the least cited was local teaching (13%). Most participants wanted support with recognising high risk presentations (60%) and providing psychological interventions (58%). The most highly requested interventions were written resources (65%), and teaching events – virtual (63%) and face-to-face (54%).

**Conclusion:**

Considering the rising prevalence of eating disorders, self-rated confidence of participants in working with young people with eating disorders was relatively low. Interventions can include providing summarised policy documents, simple reference resources, and targeted teaching. These interventions will be implemented and the survey repeated to assess impact of the intervention, with a view to repeating this cycle in order to further drive improvement.

